# Some Effects of Different Constitutive Laws on FSI Simulation for the Mitral Valve

**DOI:** 10.1038/s41598-019-49161-6

**Published:** 2019-09-04

**Authors:** Li Cai, Ying Wang, Hao Gao, Xingshuang Ma, Guangyu Zhu, Ruihang Zhang, Xiaoqin Shen, Xiaoyu Luo

**Affiliations:** 1 NPU-UoG International Cooperative Lab for Computation and Application in Cardiology, Northwestern Polytechnical University, Xi’an, 710129 China; 2 School of Mathematics and Statistics, University of Glasgow, Glasgow, G12 8QQ UK; 3 Bioengineering College, Chongqing University, Chongqing, 400030 China; 4 School of Energy and Power Engineering, Xi’an Jiaotong University, Xi’an, 710049 China; 5 School of Sciences, Xi’an University of Technology, Xi’an, 710048 China

**Keywords:** Computational biology and bioinformatics, Mathematics and computing

## Abstract

In this paper, three different constitutive laws for mitral leaflets and two laws for chordae tendineae are selected to study their effects on mitral valve dynamics with fluid-structure interaction. We first fit these three mitral leaflet constitutive laws and two chordae tendineae laws with experimental data. The fluid-structure interaction is implemented in an immersed boundary framework with finite element extension for solid, that is the hybrid immersed boundary/finite element(IB/FE) method. We specifically compare the fluid-structure results of different constitutive laws since fluid-structure interaction is the physiological loading environment. This allows us to look at the peak jet velocity, the closure regurgitation volume, and the orifice area. Our numerical results show that different constitutive laws can affect mitral valve dynamics, such as the transvalvular flow rate, closure regurgitation and the orifice area, while the differences in fiber strain and stress are insignificant because all leaflet constitutive laws are fitted to the same set of experimental data. In addition, when an exponential constitutive law of chordae tendineae is used, a lower closure regurgitation flow is observed compared to that of a linear material model. In conclusion, combining numerical dynamic simulations and static experimental tests, we are able to identify suitable constitutive laws for dynamic behaviour of mitral leaflets and chordae under physiological conditions.

## Introduction

Because mitral valve (MV) has a very complex tissue structure, any change or loss of its structure will lead to valve diseases. In recent years, valve disease have become one of the major cardiovascular diseases^1^. It is estimated that 850000 patients will be treated with valve replacement by 2050^2^. It is being recognized that the mathematical modelling and numerical simulation of the interaction between MV and blood flow are of great value and significance to deepen our understanding of valve-related diseases and treatment^3–6^.

Since early of the 19th century, researchers began to study MV geometric features and have made great progress. Different types of MV model have been developed for simulating MV dynamics, such as symmetrical geometries^7–10^, and idealized parametric models^11^. The rapid development of non-invasive clinical imaging technologies, such as ultrasound, computed tomography and magnetic resonance, have allowed the construction of patient-specific MV model. For example, Lim *et al*.^12^ built an asymmetric MV model with three-dimensional (3-D) dynamic boundaries and non-linear pressure loadings over the whole cardiac cycle based on *in-vivo* experimental data. Wenk *et al*.^13^ developed a finite element (FE) model consisting of the left ventricle, the MV leaflet and chordae tendineae using magnetic resonance images of sheep. Wang *et al*.^14^ reconstructed a patient-specific MV geometry using a multi-slice CT scan with detailed leaflet thickness and chordae tendineae structure. Ma *et al*.^15^ and Gao *et al*.^16^ developed MV models based on magnetic resonance images with fluid-structure interaction (FSI). In a recent study, Toma *et al*.^17^ constructed a MV model based on a sheep *μ*CT image data.

Early mechanical studies considered MV material as a linear elastic material^18–20^ due to its simplicity. However, various mechanical stretching experiments have shown that MV leaflets have characteristics of hyperelasticity and anisotropy^21–26^. May-newman and Yin^27^ described the nonlinear mechanical behaviors of porcine MV leaflet, and they demonstrated that the constitutive law could be derived from the hyperelastic framework under the assumption of material incompressibility and transverse isotropy. Prot *et al*.^28^ proposed two transversely isotropic hyperelastic laws for MV leaflet, and their results showed that their constitutive laws could be used to describe the mechanical properties of the normal and pathological mitral valve. Wang *et al*.^14^ employed a similar constitutive law by considering two family collagen fibers and associated dispersion with parameters estimated from *ex vivo* experiemental data. Gao *et al*.^16,29^ studied MV dynamics using a transverse isotropic strain-invariants law. There are many other forms of constitutive laws for MV leaflets existed in the literature, however, there is no agreement on which law best characterizes MV properties and predicts its dynamics.

Modelling MV dynamics are often based on structure-only models using finite element method (FEM)^12,28,30^. In the structure-only models, the transvalvular pressure load is usually directly applied to the MV leaflets as the boundary conditions. FEM is usually used for numerical implementation to study the normal MV dynamics^7^, diseased MV^8,31^, the postoperative repair^31,32^, etc. n a series of studies. Lee *et al*.^33,34^ have developed a micro- and macro- anatomically accurate MV FE model by incorporating fiber microstructure and interstitial cellular activities to study MV dynamics and facilitate treatment of diseased MV in a multiscale perspective. Besides, there were some FEM models of MV models by Wang^14^ and Prot *et al*.^21,22,28^. For recent reviews, readers are referred to^5,6^.

Physiological loading of the MV means that fluid-structure interaction (FSI) is also required to describe the MV dynamics. FSI models of MV have also been developed for decades^16,19,29,35–37^ by taking account of not only the MV structure, but also the flow field of the blood. Arbitrary Lagrangian-Eulerian (ALE) method has been most widely used to address FSI problems. For example, Dimasi *et al*.^38^ used *in vitro* and ALE FSI modeling to simulate the function of a bileaflet mechanical valves in normal and stenotic conditions with symmetric and asymmetric leaflet opening. However, ALE usually requires mesh regeneration to deal with large structural deformation, which would lead to computational difficulties when dealing with the MV dynamics. To overcome such difficulty, immersed boundary (IB) method^39^ and fictitious domain method^40^ have been developed. For example, Hart *et al*.^41^ and Loon *et al*.^42^ simulated valvular function using the fictitious domain method to analyze the coupling effects of the blood and the valve. Toma *et al*.^17^ studied the chordal forces using an ovine FSI MV system implemented with smoothed particle hydrodynamics, and validated with an advanced *in vitro* system. Our group has been developing FSI MV models using the IB approach over a decade. Watton *et al*.^43^ used a classical IB method to model a prosthetic bileaflet valve, which agreed well with the numerical results of a commercial software ANSYS. They also studied the effects of left ventricular motion on the MV dynamics^19^. Using an IB method, Luo *et al*.^44^ studied the effects of valve dynamics including the bending stiffness of the valve leaflets and artificial chordae tendineae. Ma *et al*.^15^ used a realistic MV model reconstructed from *in vivo* magnetic resonance imaging to study MV dynamics. Because soft tissue in general is anisotropic, hyperelastic and nearly incompressible, Boffi *et al*.^45^ developed an immersed boundary method with finite element discretization for the structure part. Griffith and Luo^46^ developed a hybrid approach by discretizing the incompressible Navier-Stokes equation by finite difference and the immersed structure by finite element (IB/FE). The advantage of using FE is that hyper-elasticity can be incorporated with experimentally characterized constitutive laws, which can be difficult in classical IB methods in which solid is represented by pseudo fibers^39,44^. By employing the IB/FE approach, Gao *et al*.^16^ simulated MV dynamics using a MV geometry reconstructed from *in vivo* magnetic resonance images, and further extended to a coupled MV and left ventricle^29^. In a recent study, Liu *et al*.^47^ studied the energy budget in an IB/FE MV model after a careful verification with the commercial FEM software ABAQUS.

Recent experimental or computational studies have found that the chordae tendineae plays an important role in the realization of MV function^17,48–50^. Therefore, it is necessary to incorporate suitable mechanial responses of chordae tendineae into MV models, particularly since most of the studies have used linear material models for chordae tendineae^7,9,10^. There were a few studies on the mechanical properties of the chordae tendineae using uniaxial tensile testing, on leaflet type^51^, insertion position^52^ or chordal size^53^. These studies showed that the chordae tendineae exhibits nonlinear mechanical properties and the thicker chords (strut chordae) are more extensible with lower modulus than the thinner chords (marginal chordae). However, recent uniaxial experiments with more accurate digital image tracking system from Sun’s group^51,54^ showed that there was no significant difference in tangent modulus between different kinds of chordae.

Although tensile tests of MV and chordae are important, given that physiological loading condition is dynamic and involves FSI, it is also important to evaluate the mechanical responses using various different constitutive laws with a dynamic mitral FSI system. To our best knowledge, this approach has not been fully exploited. In this study, we aim to compare three different constitutive laws for MV leaflets and two constitutive laws for chordae tendineae using an IB/FE FSI MV model developed earlier^16^, and to identify suitable constitutive laws that gives the optimal performance of MV dynamics.

## Results

### The effects of different constitutive laws of MV leaflets

With the same linear elastic constitutive law of chordae tendineae, cases M1, M2 and M3 are used to describe the mechanical properties of valve leaflets. For all three cases, the MV is fully opened at *t* = 0.1 *s* with a pressure gradient of 10 mmHg; at *t* = 0.22 *s*, the mitral valve is just closed with a pressure difference of around 80 mmHg; and at *t* = 0.35 *s*, the MV is fully loaded, the pressure gradient reaches 150 mmHg. Table 1 shows the average and maximum displacements with three constitutive laws at fully-opened and fully-loaded states. We can see that there are some differences among the three cases. For example, the average displacement from case M3 is the largest when fully opened, while that of case M1 is the largest when fully loaded. This indicates that different leaflets constitutive laws would affect MV dynamics with FSI.

**Table 1:** The average and maximum displacements.

Constitutive law	Average displacement (cm)		Maximum displacement (cm)	
	Fully opened	Fully loaded	Fully opened	Fully loaded
M1	0.16 ± 0.30	0.26 ± 0.37	1.21	1.59
M2	0.14 ± 0.24	0.20 ± 0.31	1.13	1.54
M3	0.20 ± 0.36	0.25 ± 0.38	1.38	1.52

**Figure 1:**
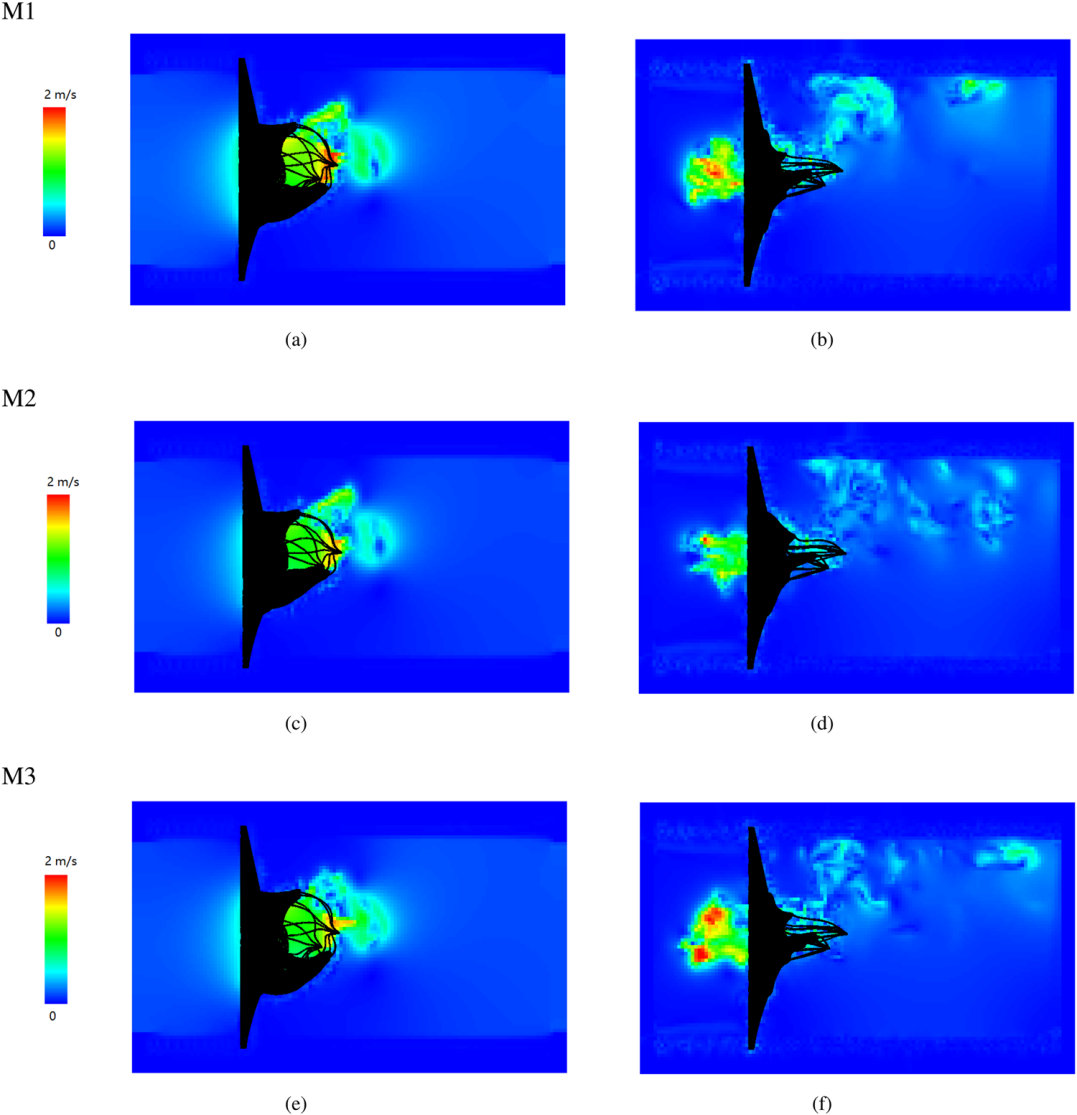
The fluid velocity field with three leaflet constitutive laws at fully-opened and just-closed states: Fully-opened (panels a,c and e), just-closed (panels b,d and f).

Figure 1 shows the velocity fields at fully-opened and just-closed states using the three constitutive laws of leaflets, respectively. It can be found that at fully-opened state (Fig. 1(a,c,e)), a strong jet is formed toward the outlet (the left ventricle side). When the MV is just closed (Fig. 1(b,d,f)), the MV leaflets prevent further blood flowing back into the left atrium side with a clear closure regurgitation, especially in cases M1 and M3. Although the general flow fields in the three cases are similar, there are some minor differences. For example, the jet in case M1 at fully-opened state is stronger than the other two cases, while the closure regurgitation flow is stronger in case M3 than other two cases. The peak jet velocity at different times are given in Table 2. Slightly lower peak velocities can be found in case M2.

**Table 2:** Peak velocity of transvalvular flow.

Constitutive model	Maximum velocity of flow field (m/s)		
	fully opened	just closed	fully loaded
M1	2.0	2.3	0.7
M2	2.0	1.9	0.7
M3	2.1	2.4	0.9

**Figure 2:**
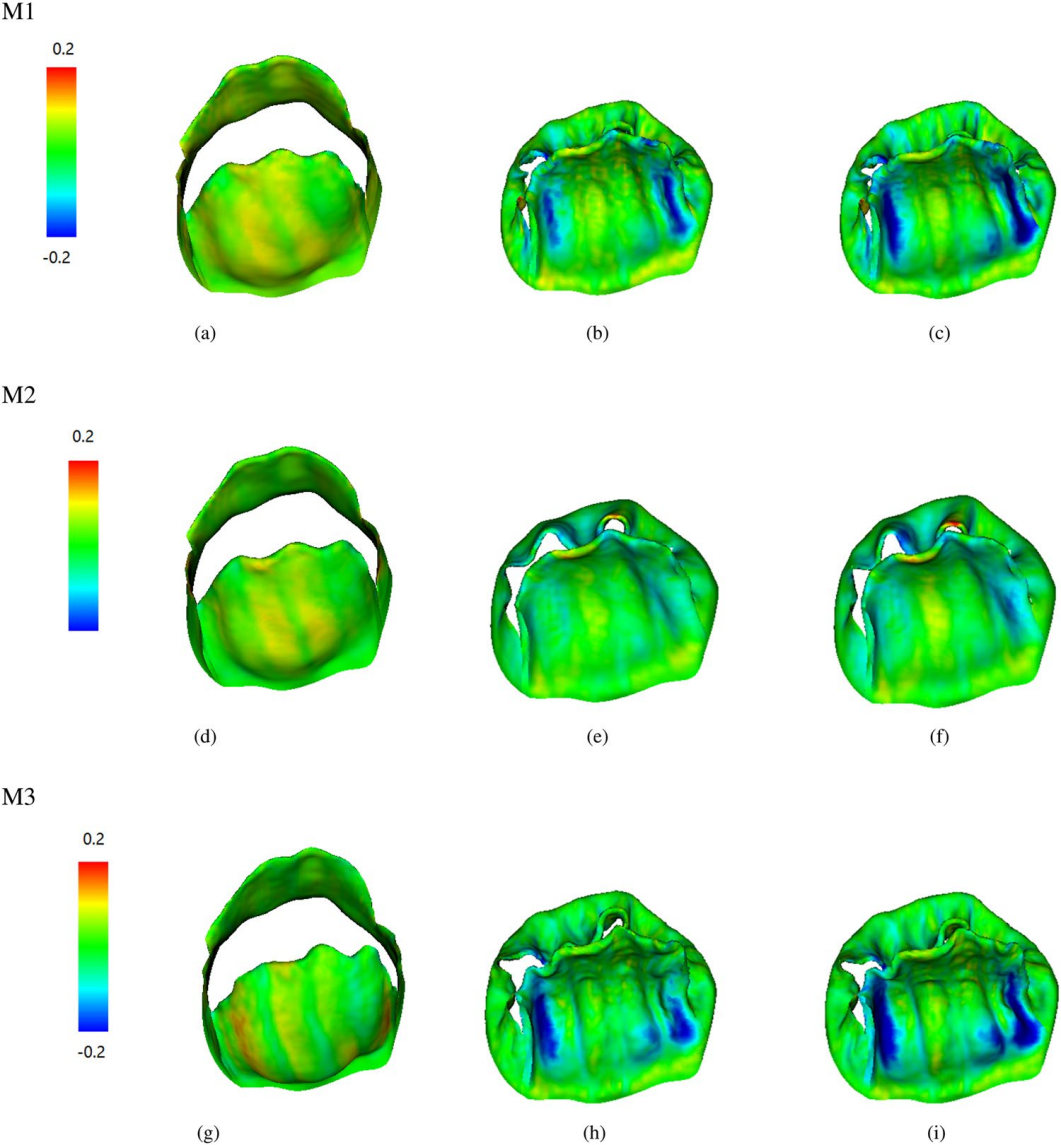
Fiber strain distributions of three constitutive laws of valve leaflet: Fully-opened (panels a,d and g), just-closed (panels b,e and h), and fully-loaded (panels c,f and i).

**Figure 3:**
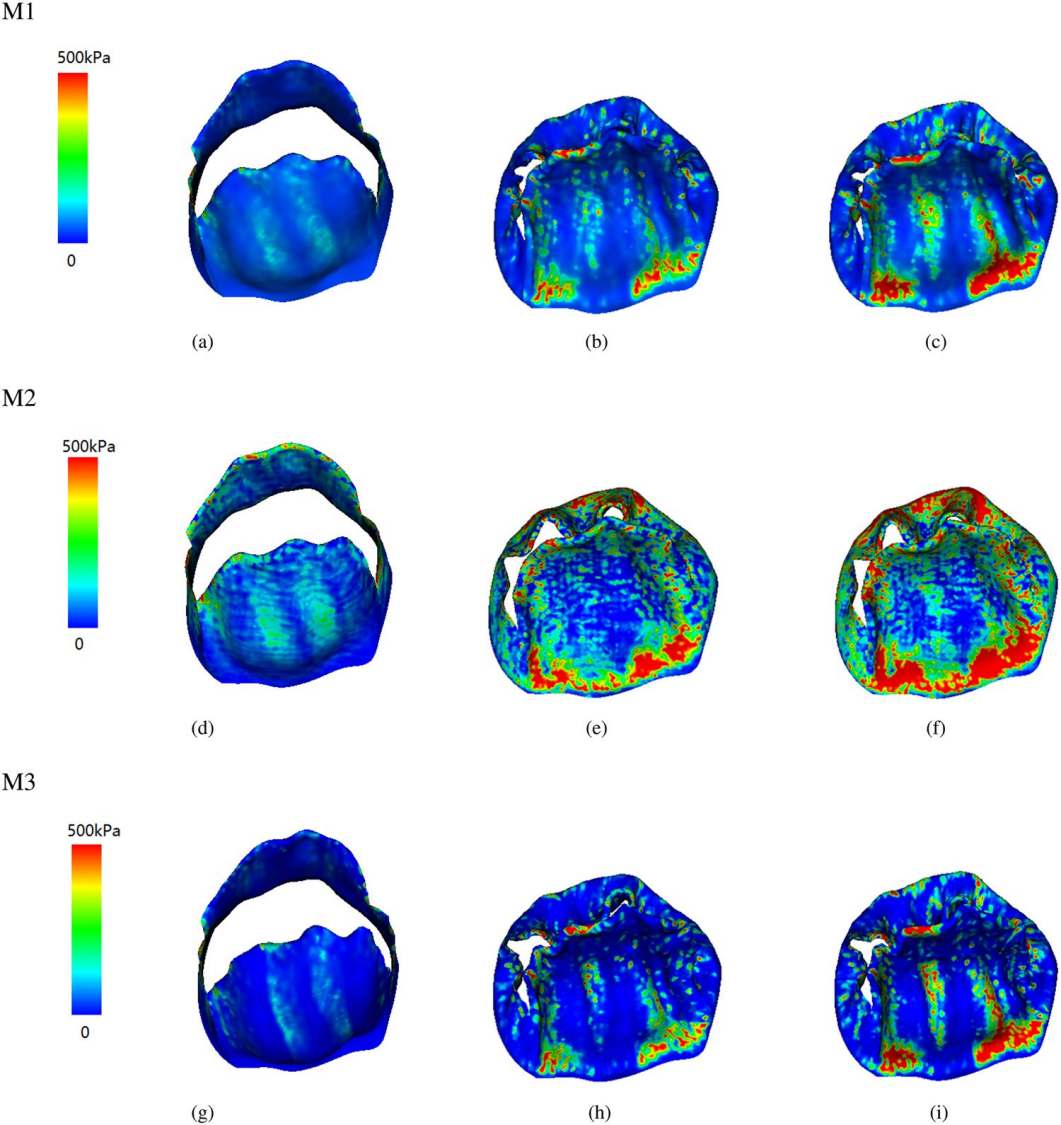
Fiber stress distributions of three constitutive laws of valve leaflets: Fully-opened (panels a,d and g), just-closed (panels b,e and h), and fully-loaded (panels c,f and i).

**Figure 4:**
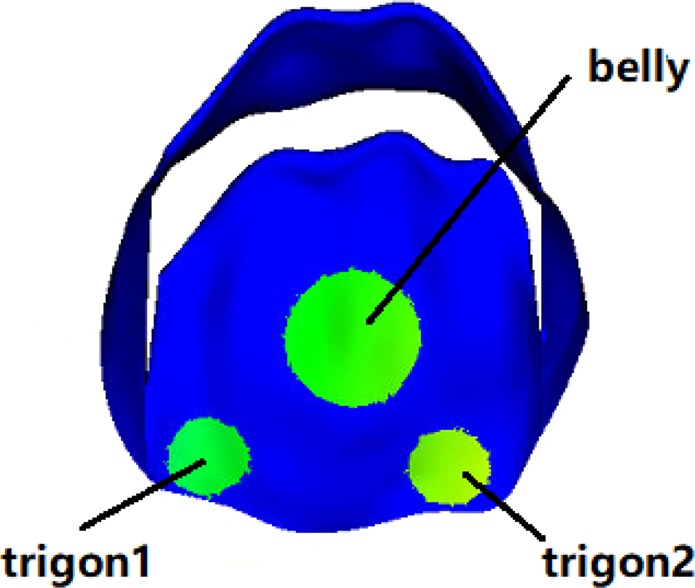
Three predefined local regions in the anterior leaflet for stress and strain calculation.

Figures 2 and 3 show the fiber strain and stress distributions. When the MV leaflets are fully opened, both the anterior and posterior leaflets are stretched along the fiber direction with low stress due to low diastolic loading. With increased transvalvular pressure, the valve leaflets start to close. When the valve is fully loaded, regions near commissures are highly compressed in cases M1 and M3, but not in case M2. The MV leaflets are further pushed towards the left atrium side with increased pressure, better closer configurations are achieved in cases M1 and M3 with smaller orifice area compared to case M2 at fully-loaded state. When the MV is closed, all cases show high stress concentration near the annulus region, the stress level in case M2 is higher than the other two cases. In order to further analyze fiber strain and stress of leaflets, we define three local regions at the anterior leaflet: two trigones and one belly region, as shown in Fig. 4. The average fiber stress and strain of these regions are summarized in Tables 3 and 4, respectively. When the MV is fully opened, the stress in the belly region is greater than that in the trigone regions; when the MV is just closed, the stress of leaflets begin to increase with much higher values in the trigones than that in the belly region; the stresses of the MV continue to increase until the MV is fully loaded. We note that at fully-opened state, all three cases experience similar stress level, while at just-closed and fully-loaded states, case M3 seems experiencing lower stress level compared with other two cases which may be caused by higher level compression in the leaflets (Fig. 2(h)).

**Table 3:** Average stresses along fiber direction on three local regions.

Model	Average stress along fiber direction (kPa)								
	Fully opened			Just closed			Fully loaded		
	M1	M2	M3	M1	M2	M3	M1	M2	M3
Trigon 1	34	28	6	130	180	89	255	325	238
Trigon 2	18	18	12	142	213	110	280	439	260
The belly region	52	59	16	78	147	62	127	283	124

**Table 4:** Average strains along fiber direction on three local regions.

Model	Average strain along fiber direction								
	Fully opened			Just closed			Fully loaded		
	M1	M2	M3	M1	M2	M3	M1	M2	M3
Trigon 1	0.10	0.10	0.10	0.09	0.09	0.08	0.07	0.09	0.07
Trigon 2	0.10	0.10	0.10	0.09	0.10	0.08	0.08	0.09	0.07
The belly region	0.10	0.12	0.10	0.10	0.10	0.10	0.10	0.12	0.10

**Figure 5:**
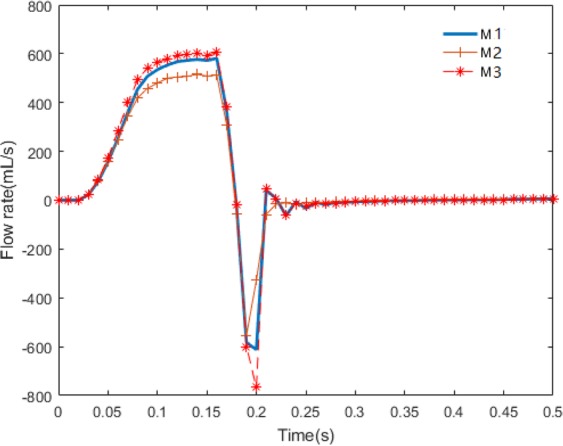
Flow rates with three material models for MV leaflets.

The transvalvular flow rates for all three cases are shown in Fig. 5. The flow rate of case M3 is higher than cases M1 and M2 in the diastolic filling phase (before 0.17 s), with M2 the lowest. When the MV begins to close, the flow rate decreases to a negative value when the closure regurgitation occurs. The regurgitation flow rates of cases M1 and M2 are similar, but much larger in case M3. Finally, the MV flow rate gradually returns to zero when the MV is fully closed. The regurgitation flow during MV closure are listed in Table 5. The results are consistent with the values from our previous work^16^. It can be found case M2 has the smallest regurgitation closing flow, while highest in case M3.

**Table 5:** Regurgitation flow (mL) during MV closure.

	M1	M2	M3
Regurgitation flow	11.5741	8.7164	13.2082

We further calculate the orifice area (OA) at fully-opened and just-closed states. In general, the higher the OA, the smaller the energy loss^55^. To calculate OA, the leaflet free-edges are first projected to the annular plane, and then the enclosed area by the project boundaries is considered to be the real OA. Table 6 shows that case M1 has the largest OA at fully-opened state and smallest OA at just-closed state, which indicates the impedance of case M1 in the diastolic filling is lower compared to other two cases and is closed more tightly in systole. The OA values from the three cases at fully-opened state are also within the interval (4–6 cm^2^) given by Luo *et al*.^44^.

**Table 6:** Orifice area (cm^2^) at different times.

	M1	M2	M3
fully-opened	4.956	4.810	4.828
just-closed	0.953	1.132	1.341

In summary, of all three models, the constitutive law M2 has the lowest leaflet displacements, lowest peak velocities at fully-opened and closed state, highest fiber stress at fully-loaded state, and lowest OA when fully-opened and the larger OA at closure. The constitutive law M1 achieves largest OA at fully-opened state and lowest OA at fully-closed state, and smaller regurgitation closure flow. Therefore, the constitutive law M1 is deemed more suitable for predicting MV dynamics with FSI. In the next section, we will use the constitutive law M1 for MV leaflets to study the effects of two different material models of the chordae tendineae on MV dynamics.

### The effects of different constitutive models of the chordae tendineae

Table 7 gives the displacements with different chordae constitutive laws. The average displacements are almost the same at fully-opened and fully-loaded states, while the maximum displacement from the linear model is slightly larger than that of the exponential model at fully-opened and -closed states.

**Table 7:** Average and maximum displacements from two chordae tendineae models.

Type of chordae tendineae constitutive law	Average displacement (cm)		Maximum displacement (cm)	
	Fully opened	Fully loaded	Fully opened	Fully loaded
Liner law	0.16 ± 0.30	0.26 ± 0.37	1.21	1.59
Exponential law	0.16 ± 0.29	0.25 ± 0.38	1.14	1.57

**Figure 6:**
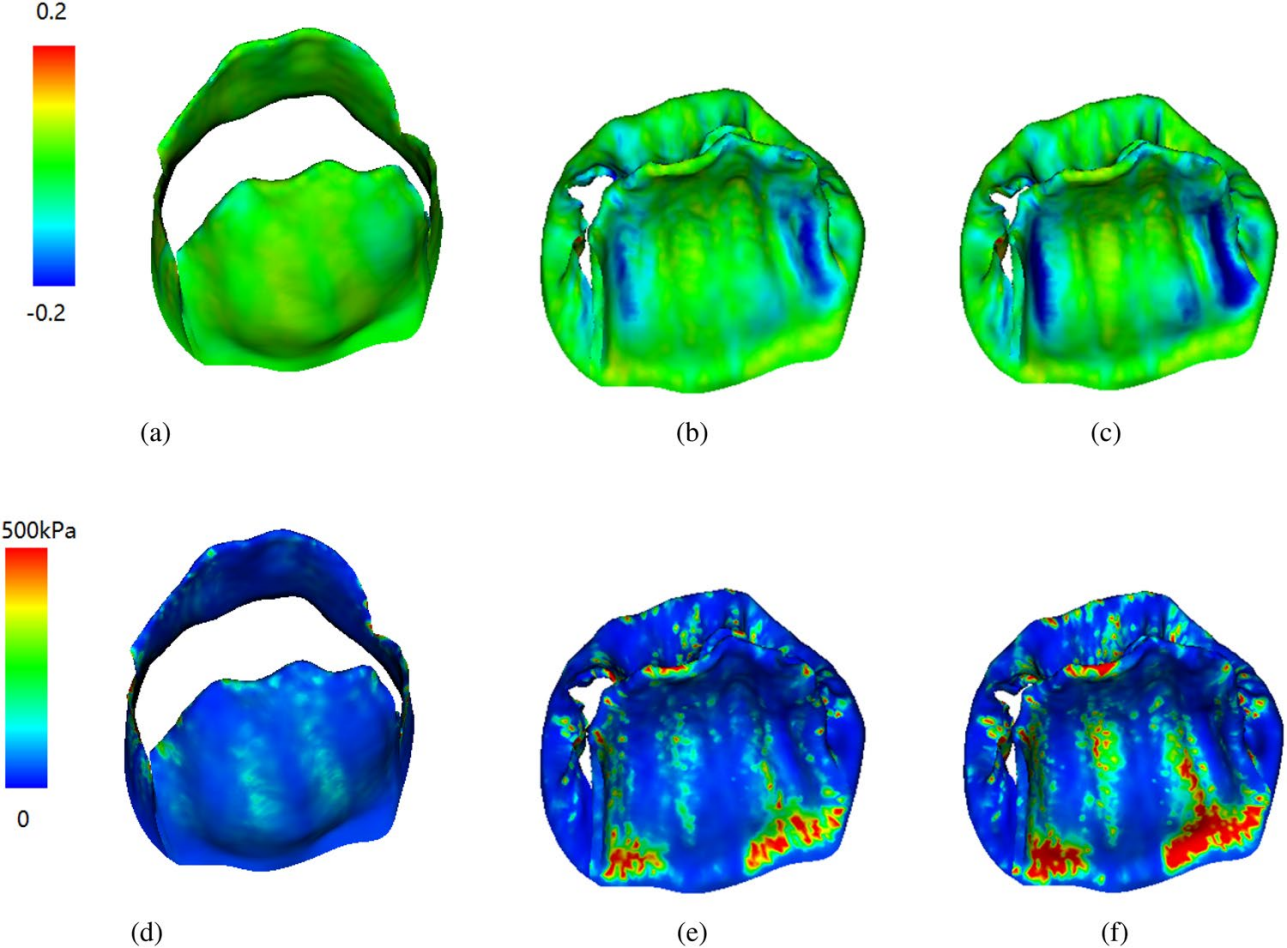
Strain and stress distributions with an exponential constitutive law of the chordae tendineae. Fully-opened (panels a and d), just-closed (panels b and e), and fully-loaded (panels c and f).

**Figure 7:**
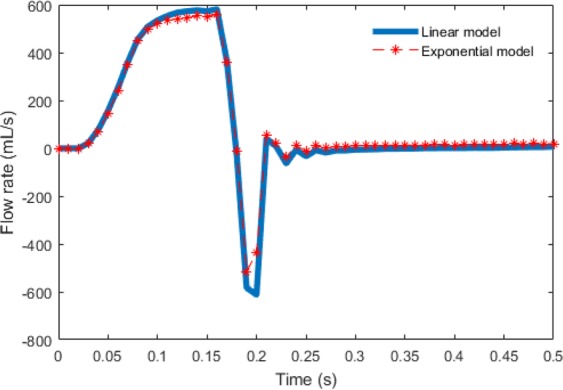
The comparison of the flow rates with chordae tendineae constitutive laws.

We summarize the fiber stress and strain results of the exponential model in Fig. 6. Comparing with the case M1 of Figs 2 and 3, we find that the stress and strain distributions of the two chordae tendineae constitutive laws are also similar. The average stress and strain of defined three regions for the exponential constitutive law are shown in Table 8. Compared to the results for the linear model (case M1), the stress level of the exponential model is slightly higher than that of the linear model. Figure 7 shows the flow rates through the MV, although slightly higher flow rate can be achieved in the linear model during diastolic filling phase (before 0.17 s), larger regurgitation closure flow exists compared to the exponential law, suggesting that the MV closes tighter when an exponential law is chosen for chordae tendineae.

**Table 8:** Average strain and stress along fiber direction on the three local regions using an exponential model for the chordae tendineae.

Model	Average strain and stress along fiber direction					
	t = 0.1 s		t = 0.22 s		t = 0.35 s	
	Strain	Stress (kPa)	Strain	Stress (kPa)	Strain	Stress (kPa)
Trigon 1	0.10	39	0.09	116	0.07	241
Trigon 2	0.09	36	0.09	147	0.08	282
The belly region	0.10	54	0.10	74	0.10	130

## Discussion

**Figure 8:**
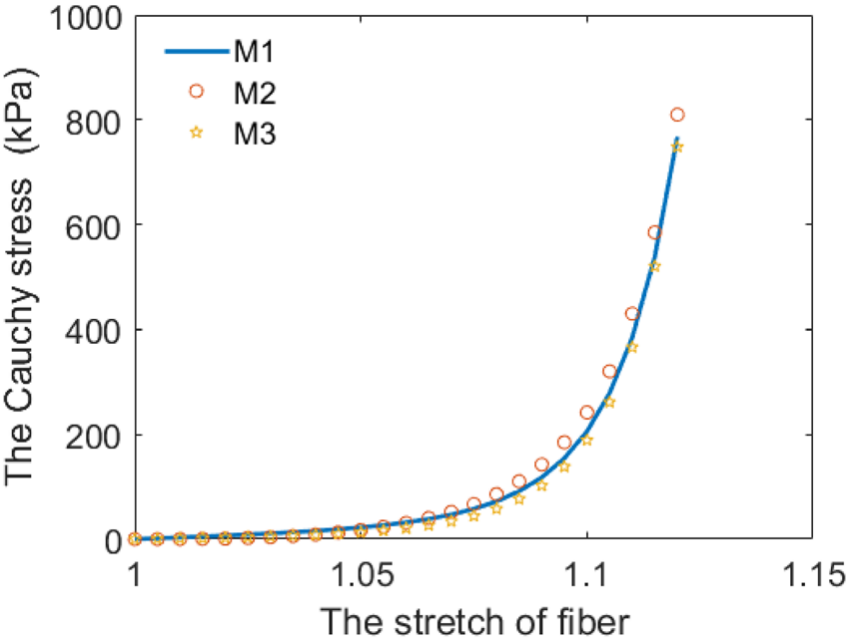
The stretch-stress relationships for the anterior leaflet along fiber direction.

**Figure 9:**
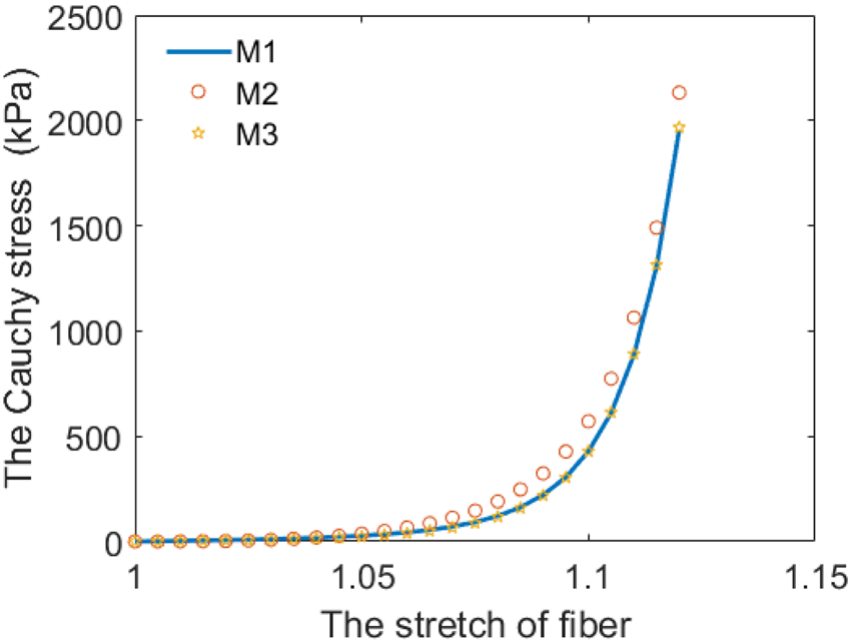
The stretch-stress relationships for the posterior leaflet along fiber direction.

In this paper, we use the IB/FE method to study the effects of different constitutive laws on MV dynamics with fluid-structure interaction. We select three different constitutive laws of MV leaflets and two material models for chordae tendineae. Parameters of different constitutive laws of leaflets are determined by matching the biaxial stretch-stress relationship along the fiber direction and the cross-fiber direction with stretch-stress relationships derived from the constitutive law M1^16^. The fitted stretch-stress curves are shown in Figs 8–9. Constitutive parameters for chordae tendineae are determined by fitting uniaxial stretching experiments of porcine chordae tendineae. All constitutive laws can capture the mechanical behavior of the MV with R-squared values similar, so then we compare the MV dynamic behaviors with different constitutive laws of the leaflet and chordae tendineae. Results show that case M1 has the largest OA at fully-opened state and the smallest OA at just-closed state, the regurgitation closing flow of case M1 and the exponential chordae tendineae model are lower than others. Our results may suggest that the combination of M1 constitutive law for the leaflet and the exponential law for chordae tendineae would be more suitable for simulating MV dynamics with FSI.

**Figure 10:**
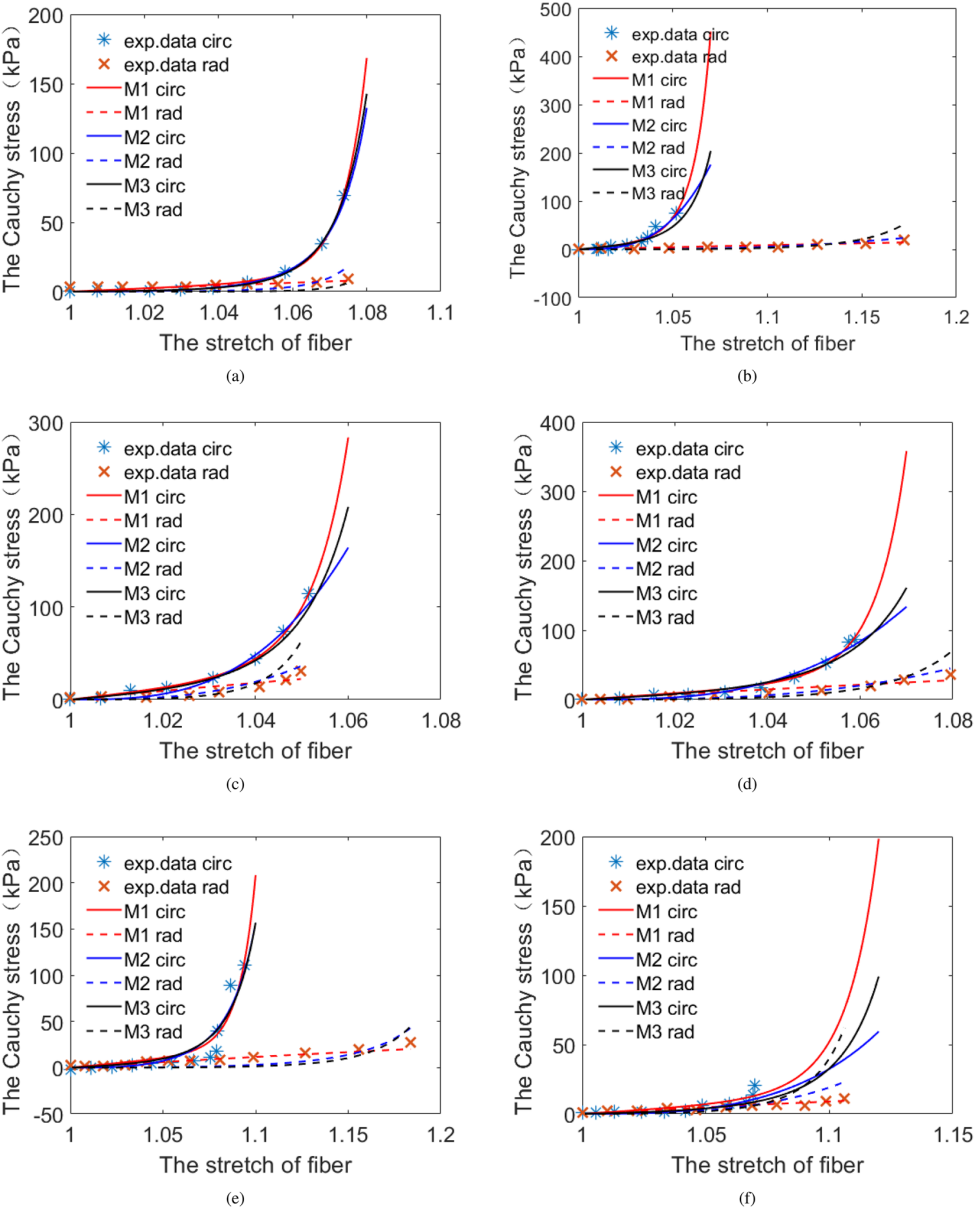
Fitting the three constitutive laws (Eq. 9) to our own bi-axial porcine experimental data for the anterior leaflet (panels a,c and e) and the posterior leaflet (panels b,d and f). Case 1 (panels a and b), Case 2 (panels c and d), Case 3 (panels e and f).

Through *in vitro*^24,25,56^ and *in vivo*^57^ studies, many researchers have also studied the mechanical properties of MV by uniaxial and biaxial stretching experiments^23,51^, and those experimental data have shown that valve leaflets and chordae tendineae have the characteristics of anisotropy and non-linearity. In papers^37,58–60^, linear elastic valve material models were used, which was impractical. Therefore, we choose fiber-reinforced constitutive laws for the MV leaflet^22,23^ in this study, a common practice in current soft tissue modelling. We further find that the three selected MV leaflets constitutive laws can fit to our own porcine MV experiments very well, as shown in Fig. 10.

The strain and stress distributions along fiber direction at different time are shown in Figs 2, 3 and 6. It can be seen that for different constitutive laws, most of the leaflets regions are tensile along the fiber direction and some of regions are compressed at closed state, which is, in general, consistent with the results of the papers^22,23^. Detailed strain and stress analysis in three regions are given in Tables 3–4 and 8. In the paper^43^, the maximum fiber strain is 0.4, which is larger than our results. However, our model prediction of the circumferential stresses range seems to agree well with the measurements from *in vivo* measurements^25,61^. For example, the circumferential stresses^25,61^ of valve leaflets range from 200 to 280 kPa at fully-loaded state. There are some differences among different constitutive laws for MV leaflets. For example, case M2 exhibits a greater stress on the posterior valves, while cases M1 and M3 experience almost the same stress level. In addition, we find that the stresses are mainly concentrated on the annulus ring and the edge of the MV leaflets during closure. When the exponential constitutive law of the chordae tendineae is used to replace the linear constitutive law, we find that the strain level of MV leaflets is larger than that of the linear model, but still within the ranges reported in^62^.

Additionally, we have compared several quantities to evaluate three different material models of MV leaflets, including the peak jet velocity, the closure regurgitation volume and the orifice area. The difference in peak velocity is minor for the three cases according to Table 2. As for the amount of regurgitation flow, case M2 has the lowest regurgitant volume and M3 has the highest regurgitant volume. Table 6 shows that case M1 has the biggest orifice area when fully opened and the smallest when just closed. The orifice area values at fully-opened state are within the interval (4–6 cm^2^) reported^44^.

## Methods

### IB/FE method

The IB/FE method developed by Griffith and Luo^46^ is employed to simulate MV dynamics in this study. The governing equations of the FSI system are

1 $$\rho (\frac{\partial {\bf{u}}}{\partial t}({\bf{x}},t)+{\bf{u}}({\bf{x}},t)\cdot \nabla {\bf{u}}({\bf{x}},t))=-\,\nabla p({\bf{x}},t)+\mu {\nabla }^{2}{\bf{u}}({\bf{x}},t)+{{\bf{f}}}^{{\rm{e}}}({\bf{x}},t),$$

2 $$\nabla \cdot {\bf{u}}({\bf{x}},t)=0,$$

3 $${{\bf{f}}}^{{\rm{e}}}({\bf{x}},t)={\int }_{E}{{\bf{F}}}^{{\rm{e}}}({\bf{X}},t)\delta ({\bf{x}}-\chi ({\bf{X}},t)){\rm{d}}{\bf{X}},$$

4 $$\frac{\partial \chi ({\bf{X}},t)}{\partial t}={\int }_{\Omega }{\bf{u}}({\bf{x}},t)\delta ({\bf{x}}-\chi ({\bf{X}},t)){\rm{d}}{\bf{x}},$$

5 $${\int }_{E}{\bf{F}}({\bf{X}},t)\cdot {\bf{V}}({\bf{X}})d{\bf{X}}=-{\int }_{E}{{\bf{P}}}^{{\rm{e}}}:\nabla {\bf{V}}({\bf{X}}){\rm{d}}{\bf{X}}.$$

where **X** = (*X*_1_, *X*_2_, *X*_3_) ∈ *E* denotes the material (Lagrangian) coordinates in the reference configuration, **x** = (*x*_1_, *x*_2_, *x*_3_) ∈ Ω denotes the Cartesian (Eulerian) coordinates. Ω ⊂ *R*^3^ denotes the physical region occupied by the fluid-structure system, and *E* ⊂ *R*^3^ denotes the region occupied by the immersed structure (such as the mitral valve, chordae tendineae, etc) in the reference configuration. *ρ* is the fluid density, *p*(**x**, *t*) is the Eulerian pressure, and *μ* is the viscosity. *χ*(**X**, *t*) ∈ Ω gives the physical position of material point **X** at time *t*. Therefore, the physical region occupied by the structure at time *t* is Ω^*e*^(*t*) = *χ*(*E*, *t*), and the physical domain occupied by the fluid at time *t* is Ω ^*f*^(*t*) = Ω − Ω^*e*^(*t*). A three-dimensional regularised delta function *δ*(**x**) = *δ*(*x*_1_)*δ*(*x*_2_)*δ*(*x*_3_) was used to describe the fluid-structure interaction, which implies that the IB/FE approach permits nonconforming discretization of the fluid and structure domains. ${{\bf{P}}}^{{\rm{e}}}=\frac{\partial \Psi }{\partial {\bf{F}}}$ is the first Piola-Kirchhoff (PK) stress tensor, which is calculated from a strain-invariant based strain energy function Ψ.

The total Cauchy stress tensor of the coupled fluid-structure system is

6 $$\sigma ({\bf{x}},t)={\sigma }^{{\rm{f}}}({\bf{x}},t)+\{\begin{array}{ll}{\sigma }^{{\rm{e}}}({\bf{x}},t) & {\rm{for}}\,{\bf{x}}\in {\Omega }^{{\rm{e}}}\\ 0 & {\rm{otherwise}}\end{array}$$

where *σ*^f^ = −*p*(**x**, *t*)**I** + *μ*[∇**u**(**x**, *t*) + (∇**u**(**x**, *t*))^T^] is the fluid-like stress tensor. **I** is the identity matrix, and *σ*^*e*^ is the elastic stress tensor, defined as

7 $${\sigma }^{{\rm{e}}}=\{\begin{array}{ll}{J}^{-1}{{\bf{P}}}^{{\rm{e}}}{{\bf{F}}}^{{\rm{T}}} & {\rm{for}}\,{\bf{x}}\in {\Omega }^{{\rm{e}}}\\ 0 & {\rm{otherwise}}\end{array}$$

where ${\bf{F}}=\frac{\partial \chi }{\partial {\bf{X}}}$ is the deformation gradient and *J* = det(**F**).

### Constitutive laws and parameters

Biological tissues usually can be modeled as nonlinear elastic materials, and their material parameters could be obtained from uniaxial or biaxial tensile testing, in which tissue samples are subjected to various stretching configurations along different directions. MV anatomy experiments show that valve tissue is basically composed of fibrous tissue^51,63^, mainly collagen and elastin, and the liquid (mainly water). At low strain, the wavy structure can be extended by relatively low stress, but with the increase of strain, the fiber straightens gradually and the overall response of the structure becomes more rigid. To determine material parameters, an inverse problem is usually formulated by minimizing the differences between the predicted stretch-stress data derived from selected constitutive laws and experimentally measured data, that is

8 $$\begin{array}{ll}{\rm{\arg }}\mathop{{\rm{\min }}}\limits_{{c}_{1},{c}_{2},{c}_{3},\cdot \cdot \cdot }\, & \sum [{(\sigma {}_{11}^{{\rm{model}}}-\sigma {}_{11}^{\exp })}^{2}+{(\sigma {}_{22}^{{\rm{model}}}-\sigma {}_{22}^{\exp })}^{2}+\cdots ]\\  & {c}_{i} > {g}_{i}\end{array}$$

where *c*_*i*_ (*i* ≥ 1) are non-negative material parameters and *g*_*i*_ are constraints of constitutive constants, i.e. >0, and *σ*^model^ is calculated from some constitutive laws, *σ*^exp^ are experimental measurements.

### The constitutive laws of the mitral valve leaflets

In this study, three fiber-reinforced strain energy functions (cases M1, M2 and M3, Eq. 9) are chosen to characterize the mechanical responses of MV leaflets, all are based on strain invariants of *I*_1_ and *I*_4_, respectively, and

$$\begin{array}{cc}{I}_{1}={\rm{tr}}{\bf{C}}, & {I}_{4}={{\bf{a}}}_{0}\cdot ({\bf{C}}{{\bf{a}}}_{0})\end{array},$$

in which **C = F**^*T*^F is the Cauchy-Green deformation tensor, **a**_0_ is the collagen fiber direction at reference state, which is an unit vector. *I*_1_ represents the overall deformation, usually is used to describe the isotropic matrix property, and *I*_4_ is the squared stretch along the collagen fiber direction.

9 $$\begin{array}{llll}{\rm{M}}1: & \Psi ({I}_{1},{I}_{4}) & = & c({I}_{1}-3)+\frac{a}{2b}(\exp [b{({I}_{4}-1)}^{2}]-1),\\ {\rm{M}}2: & \Psi ({I}_{1},{I}_{4}) & = & {\bar{c}}_{0}(\exp [{\bar{c}}_{1}{({I}_{1}-3)}^{2}+{\bar{c}}_{2}{(\sqrt{{I}_{4}}-1)}^{4}]-1),\\ {\rm{M}}3: & \Psi ({I}_{1},{I}_{4}) & = & {c}_{0}(\exp [{c}_{1}{({I}_{1}-3)}^{2}+{c}_{2}{({I}_{4}-1)}^{2}]-1),\end{array}$$

where *c*, *a*, *b*, ${\bar{c}}_{0},{\bar{c}}_{1},{\bar{c}}_{2}$ and *c*_0_, *c*_1_, *c*_2_ are the non-negative material parameters. We assume collagen fibers can only bear the load when they are stretched, but not in compression, thus *I*_4_ in Eq. 9 are replaced by ${I}_{4}^{\ast }=\,{\rm{\max }}({I}_{4},1)$. These three constitutive laws of the valve leaflets are all transversely isotropic materials, case M1 was used in paper^16^, case M2 was used in paper^21^, and case M3 was from the papers^22,28^.

The corresponding Cauchy-stress tensor for the three selected strain energy functions are

10 $$\begin{array}{rcl}{\sigma }^{{\rm{M}}1} & = & -\lambda {\bf{I}}+2{\bf{F}}\frac{\partial \Psi }{\partial {\bf{C}}}{{\bf{F}}}^{T}\\  & = & -\lambda {\bf{I}}+2c{\bf{B}}+2a({I}_{4}){\exp }^{b{({I}_{4}-1)}^{2}}\cdot {{\rm{Fa}}}_{0}\otimes {{\rm{Fa}}}_{0},\\ {\sigma }^{{\rm{M}}2} & = & -\lambda {\bf{I}}+2{\bf{F}}\frac{\partial \Psi }{\partial {\bf{C}}}{{\bf{F}}}^{T}\\  & = & -\lambda {\bf{I}}+4{\bar{c}}_{0}\exp [{\bar{c}}_{1}{({I}_{1}-3)}^{2}+{\bar{c}}_{2}{(\sqrt{{I}_{4}}-1)}^{4}][{\bar{c}}_{1}({I}_{1}-3){\bf{B}}+{\bar{c}}_{2}{(\sqrt{{I}_{4}}-1)}^{3}\frac{1}{\sqrt{{I}_{4}}}\cdot {\bf{F}}{{\bf{a}}}_{0}\otimes {\bf{F}}{{\bf{a}}}_{0}],\\ {\sigma }^{{\rm{M}}3} & = & -\lambda {\bf{I}}+2{\bf{F}}\frac{\partial \Psi }{\partial {\bf{C}}}{{\bf{F}}}^{T}\\  & = & -\lambda {\bf{I}}+4{c}_{0}\exp [{c}_{1}{({I}_{1}-3)}^{2}+{c}_{2}{({I}_{4}-1)}^{2}][{c}_{1}({I}_{1}-3){\bf{B}}+{c}_{2}({I}_{4}-1)\cdot {\bf{F}}{{\bf{a}}}_{0}\otimes {\bf{F}}{{\bf{a}}}_{0}],\end{array}$$

in which *λ* is a Lagrangian multiplier to enforce the incompressibility, and B = FF^*T*^.

Parameters for the strain energy function M1 are from Gao *et al*.^16^, which are derived from the *in vitro* testing on a healthy human MV carried out by Wang *et al*.^64^. The parameters of case M1 are shown in the Table 9. Parameters in cases M2 and M3 are determined by using a “pseudo” biaxial stretching experiments along fiber and cross-fiber directions using case M1. The *fmincon* function in MATLAB is employed to determine the parameters of constitutive laws M2 and M3 by using the Cauchy stress formulas (Eq. 10) by minimizing Eq. 8. The fitted stretch-stress curves for all three laws are given in Figs 8–9, and the estimated parameters of cases M2 and M3 are listed in Table 10, including the average errors.

**Table 9:** The constitutive parameters of M1.

Parameters	*c*(kPa)	*a*(kPa)	*b*
Anterior leaflet	17.4	31.3	55.93
Posterior leaflet	10.2	50.0	63.48

**Table 10:** The constitutive parameters of M2 and M3.

Parameters of M2	${\bar{c}}_{0}$(kPa)	${\bar{c}}_{1}$(kPa)	${\bar{c}}_{2}$	Average error
Anterior leaflet	7.69	69.42	0.0008	15.0
Posterior leaflet	13.99	80.22	0.1447	57.1
**Parameters of M3**	***c***_**0**_ **(kPa)**	***c***_**1**_ **(kPa)**	***c***_**2**_	**Average error**
Anterior leaflet	0.29	0.47	55.39	10.4
Posterior leaflet	0.44	2.58	61.46	3.6

We further fit the three constitutive laws from Eq. 9 to an *in vitro* biaxial stretching experiment on porcine MV samples. In brief, fresh porcine MV samples were harvested from a local abattoir. Specimens of MV and chordae were then dissected and stored in 4 °C phosphate buffer saline (PBS) before test and submerged in 37 °C PBS bath during test. Planar biaxial tensile test was conducted with a CellScale BioTester (Waterloo, ON, Canada) with 10 N load-cell on MV samples, while uniaxial tensile test (Instron Industrial Products, US) was carried out on chordae samples. The tissue specimens were stretched and released for 8 complete cycles for preconditioning until the load-displacement curve was visibly repeatable. Finally, the MV and chordae specimens were stretched to 1500 mN and 5 N to cover the physiological condition, respectively. The displacement and tensile forces were recorded and used for stress and strain analysis. The thickness of specimens was measured by a digital caliper (±0.01 mm) three times on random location before the test. We find that all three constitutive laws can fit the experimental data very well as can be seen from Fig. 10, among which cases 1, 2 and 3 represent the three sets of MV data from three hearts. The model M2 shows the best fitting although the difference is small. Corresponding R-squared values are reported in Tables 11–12, all have minor differences for the anterior and posterior leaflet respectively, which suggests that these selected three constitutive laws are suitable for characterizing MV leaflets properties. Thus, we will mainly compare their effects on the MV dynamics using a FSI solver in this study.

**Table 11:** R-squared values of fitting the anterior leaflet with three constitutive laws in Eq. 9 to our porcine MV experiments. SSE is the residual sum of squares, SST denotes the total sum of squares.

Anterior leaflet	R-squared = 1−SSE/SST		
	M1	M2	M3
Case1	0.9904	0.9734	0.9532
Case2	0.9810	0.9822	0.9929
Case3	0.8987	0.9056	0.8909
Average value	0.9567	0.9537	0.9457

**Table 12:** R-squared values of fitting the posterior leaflet with three constitutive laws in Eq. 9 to our porcine MV experiments. SSE is the residual sum of squares, SST denotes the total sum of squares.

Posterior leaflet	R-squared = 1−SSE/SST		
	M1	M2	M3
Case1	0.9472	0.9799	0.9561
Case2	0.9722	0.9822	0.9707
Case3	0.7742	0.8112	0.8122
Average value	0.8979	0.9244	0.9130

### The constitutive laws of the chordae tendineae

Two constitutive laws are chosen for the chordae tendineae, one is the neo-hooken material model^16^, and the second one is the exponential model^28^, they are

11 $$\begin{array}{rcl}\Psi  & = & C({I}_{1}-3),\\ \Psi  & = & {a}_{1}(\exp [{a}_{2}({I}_{1}-3)]-1).\end{array}$$

in which *C*, *a*_1_, and *a*_2_ are material constants. The Cauchy stress for the two chordae constitutive laws are

12 $$\begin{array}{rcl}\sigma  & = & -\lambda {\bf{I}}+C{\bf{B}},\\ \sigma  & = & -\lambda {\bf{I}}+2{a}_{1}\exp [{a}_{2}({I}_{1}-3)]{\bf{B}}.\end{array}$$

**Figure 11:**
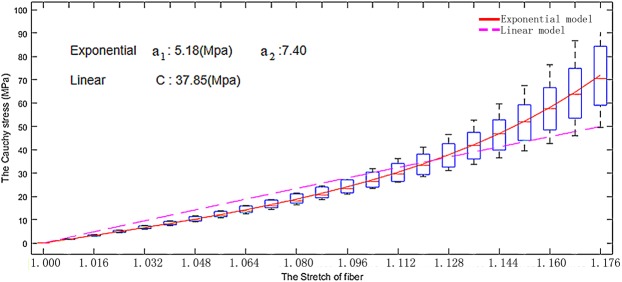
The fitted stretch-stress relationships for the chordae tendineae.

Figure 11 shows the fitted stretch-stress curves with the two chordae constitutive laws to our own uniaxial tensile testing experimental data from Dr. Ma’s Lab using porcine MV chordae tendineae. It can be found that nonlinear mechanical property of the chordae tendineae can only be better represented by the exponential law.

### The MV model and boundary conditions

**Figure 12:**
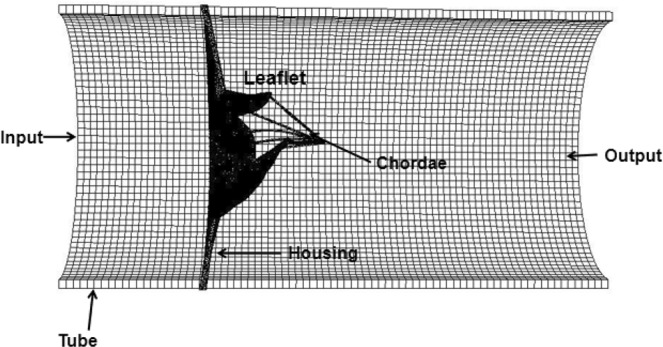
The IB/FE MV model.

The MV model is reconstructed from a cardiac magnetic resonance (CMR) imaging of a healthy volunteer, and the leaflets are reconstructed at mid-diastole, and a pseudo-chordae structure is used because the CMR imaging cannot describe the chordal structure *in vivo* due to resolution limitation. Details of the MV geometry reconstruction can be found in our previous study^16,36^. Figure 12 shows the MV with the chordae tendineae, mounted in a housing and then attached to a straight tube (length: 16 cm, radius: 3.8 cm), and immersed into a fluid domain with size 10 cm × 10 cm × 16 cm, which is discretized into 80 × 80 × 128 regular grids.

**Figure 13:**
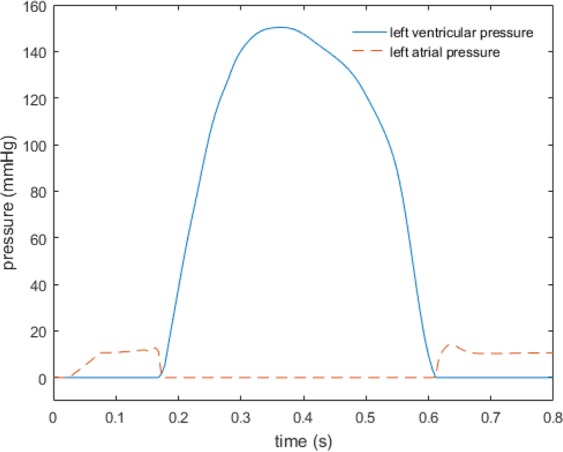
Typical human pressure profiles of MV.

An explicit version of Crank Nicolson-Adams Backward scheme is used for time stepping, which requires a relatively small time step size (10^−5^ s). The IB/FE MV model is implemented within the open-source IBAMR software framework (https://github.com/IBAMR/IBAMR). The boundary conditions are the same as in paper^16^, in brief, pressure boundary conditions are applied to the inlet of the straight tube, pressure profile is shown in Fig. 13. Zero pressure boundary conditions are applied along the rest of the boundaries of the whole computational domain. The housing and the straight tube are fixed in place. CMR measured displacements of the papillary muscles are applied to the chordae attachment points where the chordae tendineae connects the papillary muscles. Further details of the MV model implementation can be found in^16^.

### Limitations

Finally, we mention the limitations of this study. Though we have incorporated FSI and nonlinear constitutive laws for valve leaflets and chordae tendineae. We have ignored the valve-heart interactions, which will have some impact on the dynamic loading conditions^29^. In addition, our geometric structure is based on a simplified model of chordae tendineae, whereas the realistic chordae tendineae consists of marginal, strut and basal chordae tendineae^17^. We leave investigation of these effects to future research.
